# Quantity and quality of obesity-related research in Arab countries: assessment and comparative analysis

**DOI:** 10.1186/1478-4505-12-33

**Published:** 2014-07-08

**Authors:** Waleed M Sweileh, Sa’ed H Zyoud, Samah W Al-Jabi, Ansam F Sawalha

**Affiliations:** 1 Department of Pharmacology and Toxicology, College of Medicine and Health Sciences, An-Najah National University, Nablus, P.O. Box 7, Palestine; 2 Department of Clinical and Community Pharmacy, College of Medicine and Health Sciences, An-Najah National University, Nablus, P.O. Box 7, Palestine

**Keywords:** Arab countries, Bibliometric, ISI Web of Science, Obesity

## Abstract

**Background:**

Obesity is a serious worldwide medical condition, considered by some researchers as one of the most serious public health problems of the 21^st^ century. The main objective of this study was to assess the quantity and quality of obesity-related research from Arab countries and compare it with that from non-Arab Middle Eastern countries.

**Methods:**

Original scientific articles or reviews published by Arab countries about obesity up until 2011 were screened using the ISI Web of Science database. Research activity was assessed by analyzing the annual research productivity, journals names, citations, top 10 active institutions, and the contribution of each Arab country to obesity research.

**Results:**

The total number of original and review research articles published globally about obesity was 110,167. The leading country in obesity research was United States of America (42.47%). Turkey, Israel, and Iran were in the top 30 countries while Kingdom of Saudi Arabia (KSA), Egypt, and Kuwait ranked 39^th^, 43^rd^, and 47^th^, respectively. A total of 1,121 documents about “obesity” were published by Arab countries, representing 1.0% of the global research output, with 13,343 citations (average citation of 11.9 per document) and an *h*-index of 44. The Arab countries’ research output was very low until the mid-1990s and then increased steadily. Of the 1,121 documents, 107 (9.55%) were published in the *Saudi Medical Journal*. KSA, with a total of 318 publications ranked first among Arab countries in research quantity while Kuwait ranked first after adjustment based on population size. King Saud University in KSA was the most productive institution with a total of 140 documents. Compared with other non-Arab Middle Eastern countries, the research productivity from Arab countries was lower than that from Turkey, higher than that from Iran, and close to that from Israel. However, the *h*-index of documents about obesity published from Arab countries was lower than that of Turkey and Israel, but slightly higher than that from Iran.

**Conclusions:**

The present data reveals a good contribution by some Arab countries, particularly Arab gulf countries, to obesity research. More efforts are needed by other Arab countries to bridge the gap in this topic and to improve the quality of obesity-related research originating from Arab countries.

## Introduction

Obesity is a serious worldwide medical condition [1], and is considered by some researchers as one of the most serious public health problems of the 21^st^ century [2]. Several risk factors have been cited as possible causes for obesity, including the lack of physical activity, excessive eating, and endocrine and genetic predisposition [3,4]. According to the World Health Organization, there were 400 million obese people in 2005 and the number will jump to 700 million people by 2015 [5]. The Arab world is no exception and prevalence of obesity is high in the Arab world, particularly in Arab gulf countries [6-8].

Reducing obesity-related mortality and morbidity in Arab countries requires an understanding of how these various countries progress in scientific research pertaining to obesity. Such an understanding is instrumental for the development of an effective plan to respond to this global problem. Research assessment from a particular country could be achieved through bibliometric analysis in which statistical methods are implemented [9]. Bibliometrics has been applied to various diseases and is now widely accepted as a method of measuring research and literacy output in any particular research area [10,11].

The objective of this study was to analyze the quantity and quality of obesity-related research activity in Arab countries and to compare it with that from neighboring non-Arab countries. Such a study will lead to a better understanding of the current and future status of obesity research in Arab countries. The results of the study will also help health researchers to develop future research and keep the momentum of research activity in the Arab world.

## Methods

The data used in this study were based on ISI Web of Science (WoS), which is one of the world’s largest databases of peer-reviewed literature. The WoS databases provide authoritative, multidisciplinary coverage from more than 12,000 high impact research journals worldwide [12]. WoS was developed by Thomson Scientific and is well known for its annual report on journal impact factors (IFs), which is an important measure of the quality and influential power of journals. WoS is easy to use and has a simple and advanced search tool, with which a list of codes can be used to achieve the required objective of the search. In the advanced search, the WoS allows the researcher to analyze data based on several variables including types of documents, research areas, source titles, publication years, countries, and languages of published documents. In this manuscript, research areas of obesity documents were obtained directly from WoS advanced search analysis. At any point in the analysis, the WoS allows the researcher to select any item and refine the data or to select any item and exclude it from analysis. Furthermore, advanced search in WoS allows the researcher to select any key word and search for documents that have that key word in the title. Several research results can also be combined and the resultant file can be analyzed. In this manuscript, epidemiology-related documents were searched using the key words “epidemiology” or “prevalence” in the advanced search results obtained after entering the country codes and research topic code. The results of advanced search analysis can be copied and transferred to Microsoft excel for further analysis.

Detailed citation analysis of the advanced search results can also be easily obtained. A comprehensive analysis of the advantages and disadvantages of various databases, including WoS, PubMed, and Scopus, is presented by Falagas et al. [13]. In contrast to PubMed, WoS covers most scientific publications and not only medical and biomedical publications, as in the case of PubMed. Furthermore, WoS covers the oldest publications and its records go back to 1900. A major disadvantage of PubMed is the fact that it does not provide citation analysis and therefore does not allow for qualitative analysis of published literature [13]. The objective of the current study was to assess the quantity and quality of obesity-related research from Arab countries. Therefore, at least half of the objective, which is the qualitative analysis of obesity-related research, cannot be carried out by PubMed. Furthermore, although obesity is a medical subject, research about obesity could have been carried out and published in non-medical journals which make PubMed less suitable than WoS for this purpose. Actually, a quick search for PubMed using “obesity” key word yielded a lower number of publications than obtained by WoS.

In this study, all Arab countries including Kingdom of Saudi Arabia (KSA), Egypt, Jordan, Lebanon, Qatar, Bahrain, Kuwait, Morocco, Tunisia, Syrian Arab Republic (SAR), United Arab Emirates (UAE), Iraq, Sudan, Yemen, Algeria, Comoros, Djibouti, Libya, Mauritania, Oman, and Somalia, except Palestine, were used as country keys followed by “obesity” key word as a topic. We chose the key word “obesity” and excluded any other words like “overweight” because we are interested in obesity per se rather than associated terminology. The obesity key word was entered in advanced search in WoS as a search topic. The search topic indicates that the search engine in the WoS database will search for all articles in which the title or the abstract or the key words of the article have the word “obesity”. This strategy for retrieving articles from WoS has been previously used [14]. Furthermore, the WoS database has been used in at least 150 published studies to assess the quantity and quality of research output in different disciplines [14,15]. The search strategy looked like this: (CU = (Jordan) OR CU = (Iraq) OR CU = (Syria) OR CU = (Saudi) OR CU = (Kuwait) OR CU = (Egypt) OR CU = (Yemen) OR CU = (Qatar) OR CU = (Emirates) OR CU = (Bahrain) OR CU = (Oman) OR CU = (Sudan) OR CU = (Tunisia) OR CU = (Algeria) OR CU = (Lebanon) OR CU = (Libya) OR CU = (Morocco) OR CU = (Somalia) OR CU = (Djibouti) OR CU = (Comoros) OR CU = (Mauritania) AND search topic = (Obesity). Palestine was excluded from search keys because the WoS database does not recognize Palestine as an independent state yet. Furthermore, to increase the accuracy of results, research was refined and limited to original research articles and review articles because they represent the research activities, while other types of documents, such as editorials, conference proceedings, and others, were excluded. The time frame for the result was up to the year 2011. The 2012 and 2013 years were excluded because they are still open for new journal issues.

The WoS database generates a count of total number of original articles, total citations, and the value of the highly-cited index (*h*-index). The *h*-index represents the number of citations received for each of the documents in descending order, while the *h*-graph measures the impact of a set of documents and displays the number of citations per document (for example: an *h*-index of 10 means that there are 10 items that have 10 citations or more). The *h*-index was originally developed as a measure of qualifying research performance [16,17]. Publication activity was adjusted for Arab countries based on population size and therefore the total number of published documents per million inhabitants was presented. The number of population for each country was obtainedfrom the online databases of the World Bank [18]. The collected data were used to generate the following bibliometric indicators which have used in previous similar publications [19,20]: (a) total and trends of contributions to obesity research during all previous years up to the set date of data analysis (December 31^st^, 2011); (b) Arab countries research productivity and collaboration patterns; (c) journals in which Arab country researchers published; and (d) the number of citations received by the publications.

### Ethical approval

The Institutional Review Board (IRB) at An-Najah National University does not require submission of an IRB application for such a study. The IRB considered that there is no risk for human subjects in such publications since the data are based on published literature and did not involve any interactions with human subjects.

### Statistical analysis

Data from WoS were exported to Microsoft Office Excel® and then transferred to the Microsoft word program. The measurements of bibliometric analysis (e.g., countries, cited articles, institutions) were converted to the rank order using the standard competition ranking; we took into consideration the top 10 ranking in each item. If the measurements of bibliometric analysis have the same ranking number, then a gap is left in the following ranking numbers. The journalIFs were evaluated using the Journal Citation Report (Web of Knowledge) 2012 science edition by Thomson Reuters (New York, NY, USA).

## Results

The total number of documents retrieved from WoS using the methodology stated and without specifying the name of any country was 110,167. This number represents the global research productivity (original research articles and reviews) about obesity as a “research topic” up to the year 2011. Global research productivity in obesity was lower than that for hypertension (202,838 documents) and that for diabetes mellitus (126,187 documents). The leading countries in obesity research were the USA (46,783; 42.47%) followed by England (8,099; 7.35%) and Japan (5,857; 5.32%). Worldwide, Turkey, Israel, and Iran were among the top 30 countries in obesity research while KSA, Egypt, and Kuwait ranked 39^th^, 43^rd^, and 47^th^ respectively. More than 95% of worldwide obesity documents were written in English and less than 5% were written in 22 other languages, mainly German and French. The annual productivity of research about obesity remained low until 1990, after which a steady and sharp increase in obesity research was observed globally. More than 50% of published obesity documents were in the research areas of endocrinology, nutrition, general/internal medicine, and public health. The most productive institutions of obesity research were Harvard University, University of Minnesota, and University of Texas with a total of 2,886, 1,401, and 1,230 documents, respectively.

When the same methodology was applied using the list of the 21 Arab countries, 1,121 documents were retrieved. Therefore, obesity-related research from Arab countries represents 1.0% of the worldwide research productivity. As a comparison, Arab countries have published 2,022 documents in diabetes mellitus and 1,966 documents in hypertension. Of the 1,121 documents about obesity published by Arab countries, 1,077 (96.08%) were written in English, while the remaining 44 (3.92%) documents were published in German and French. A total of 193 (17.2%) obesity-related documents published from Arab countries were open access while the remaining (928; 82.8%) were not. General/internal medicine was the main research area of the 1,121 published documents followed by endocrinology/metabolism and nutrition/didactics. The first article about obesity co-authored by an Arab researcher was published in 1980 in the *Archives of Disease in Childhood* and the title of the article was: “*Excessive carbohydrate intake in pregnancy and neonatal obesity –study in Cap-Bon, Tunisia*” [21].

**Figure 1:**
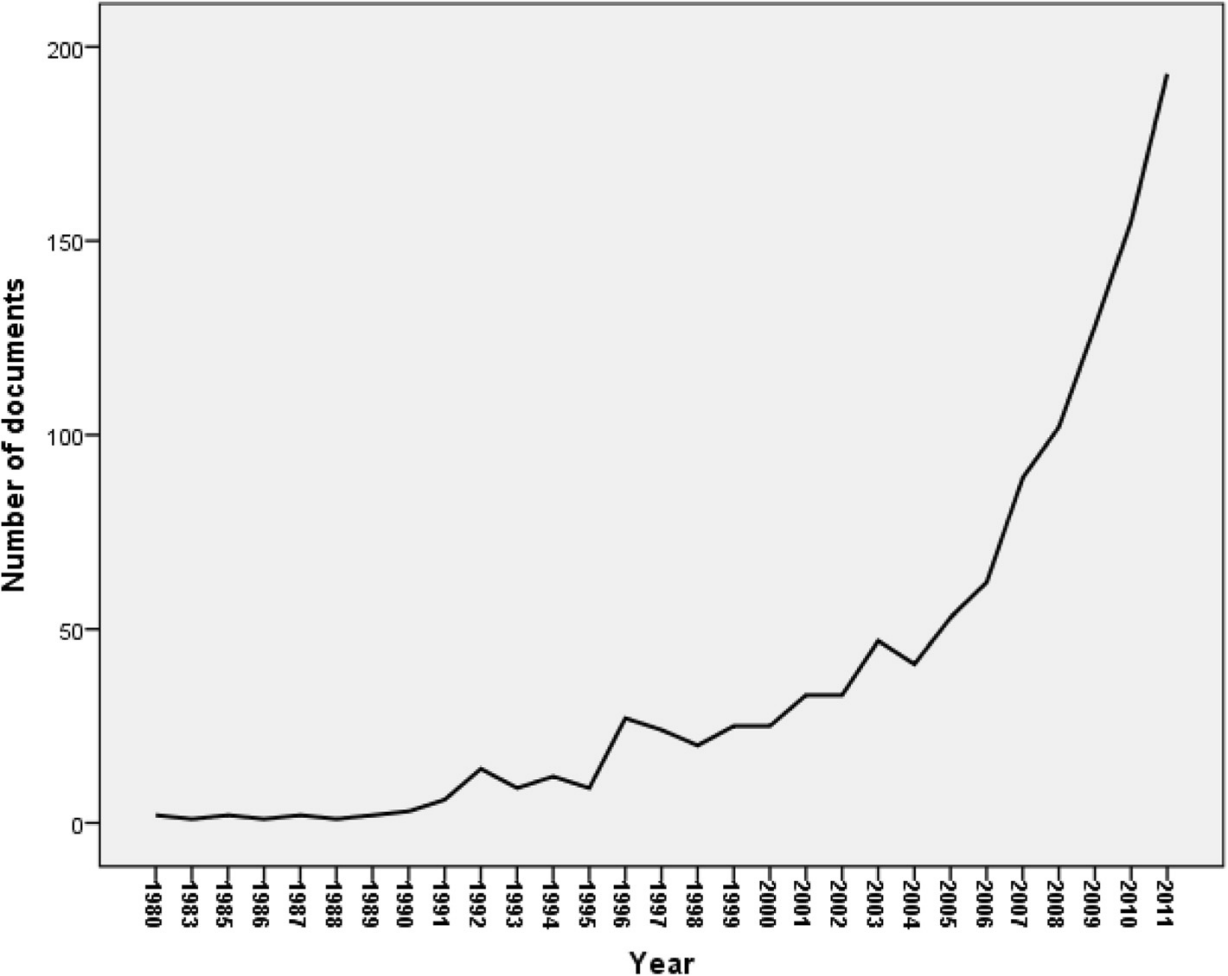
Growth of obesity research from Arab countries until 2011.

The annual number of documents published from Arab countries indicated that research activity about obesity remained low until the mid-1990s and showed a steady increase after the year 2000. More than 50% of documents about obesity were published during 2008 to 2011 (Figure 1). When retrieved data was analyzed by country, KSA (318; 28.37%) had the highest quantity of documents followed by Egypt (193; 17.22%) and Kuwait (132; 11.78%). Slightly less than half (48.5%) of obesity documents from Arab countries came from three Arab gulf countries, particularly KSA, Kuwait, and UAE. No data related to obesity was found from Somalia, Djibouti, Mauritania, and Comoros (Table 1). Standardization of research activity using number of population showed that Kuwait (40.62 documents per million inhabitants) was the top productive Arab country followed distantly by Bahrain (34.14 documents per million inhabitants) and Qatar (20.97 documents per one million inhabitants, respectively) (Table 1).

**Table 1:** Contribution of each Arab country to published obesity documents

**Country**	**Number of documents**	**Number of published documents per million inhabitants**
	**n(%) = 1,121 (%)**^**a**^	
Kingdom of Saudi Arabia	318 (28.37)	11.24
Egypt	193 (17.22)	2.39
Kuwait	132 (11.87)	40.62
Tunisia	109 (9.72)	10.11
United Arab Emirates	95 (8.48)	10.32
Lebanon	73 (6.51)	16.50
Jordan	61 (5.44)	9.65
Morocco	49 (4.37)	1.51
Bahrain	45 (4.01)	34.14
Qatar	43 (3.84)	20.97
Oman	36 (3.21)	10.86
Algeria	23 (2.05)	0.60
Libya	14 (1.25)	2.27
Iraq	13 (1.16)	0.40
Sudan	10 (0.89)	0.27
Yemen	7 (0.62)	0.29
Syria	5 (0.45)	0.22
Somalia	0	0
Comoros	0	0
Djibouti	0	0
Mauritania	0	0

^a^Total exceeds 100% because of overlap in some documents among more than one Arab country.

**Figure 2:**
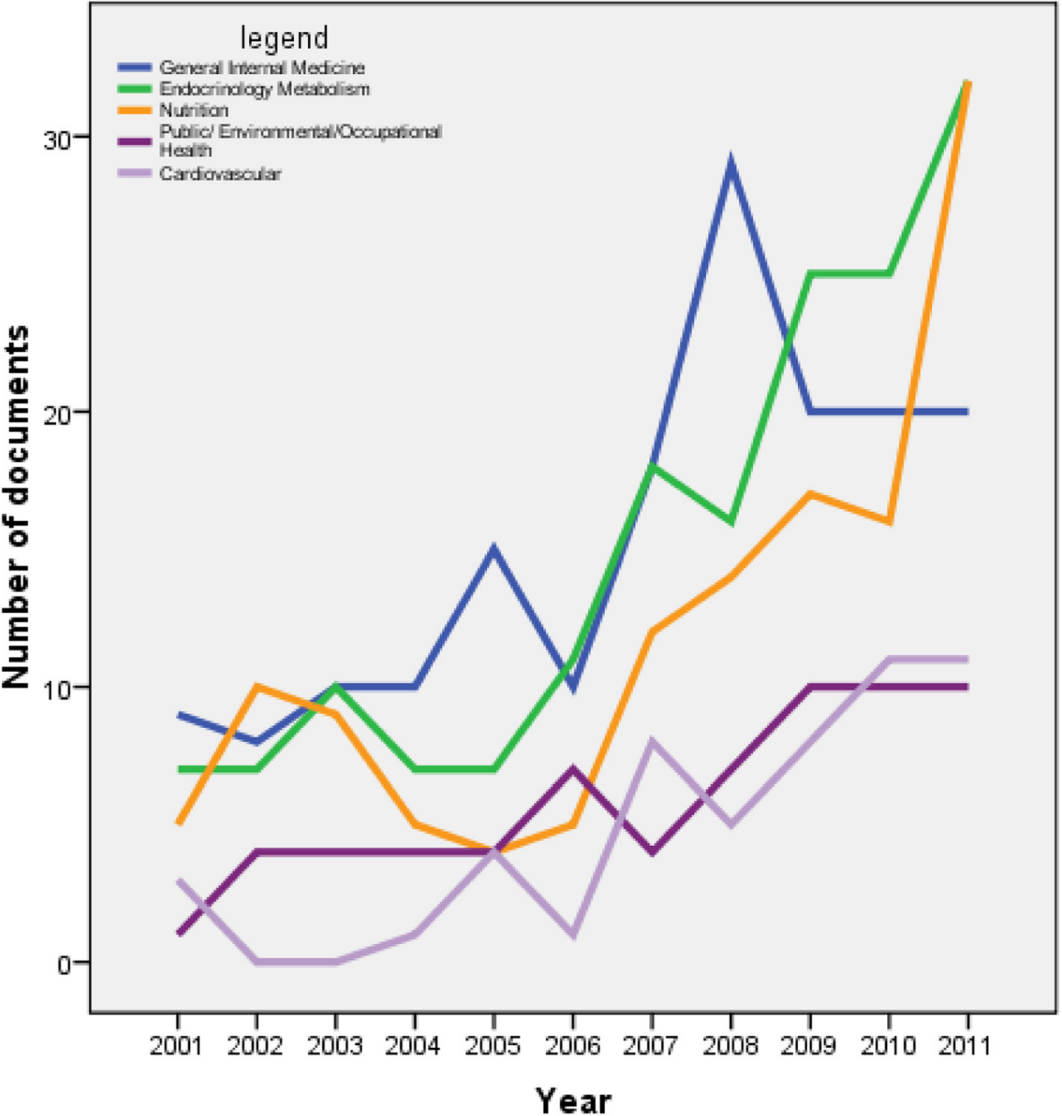
Emergence of selected obesity research areas with time in Arab countries.

**Figure 3:**
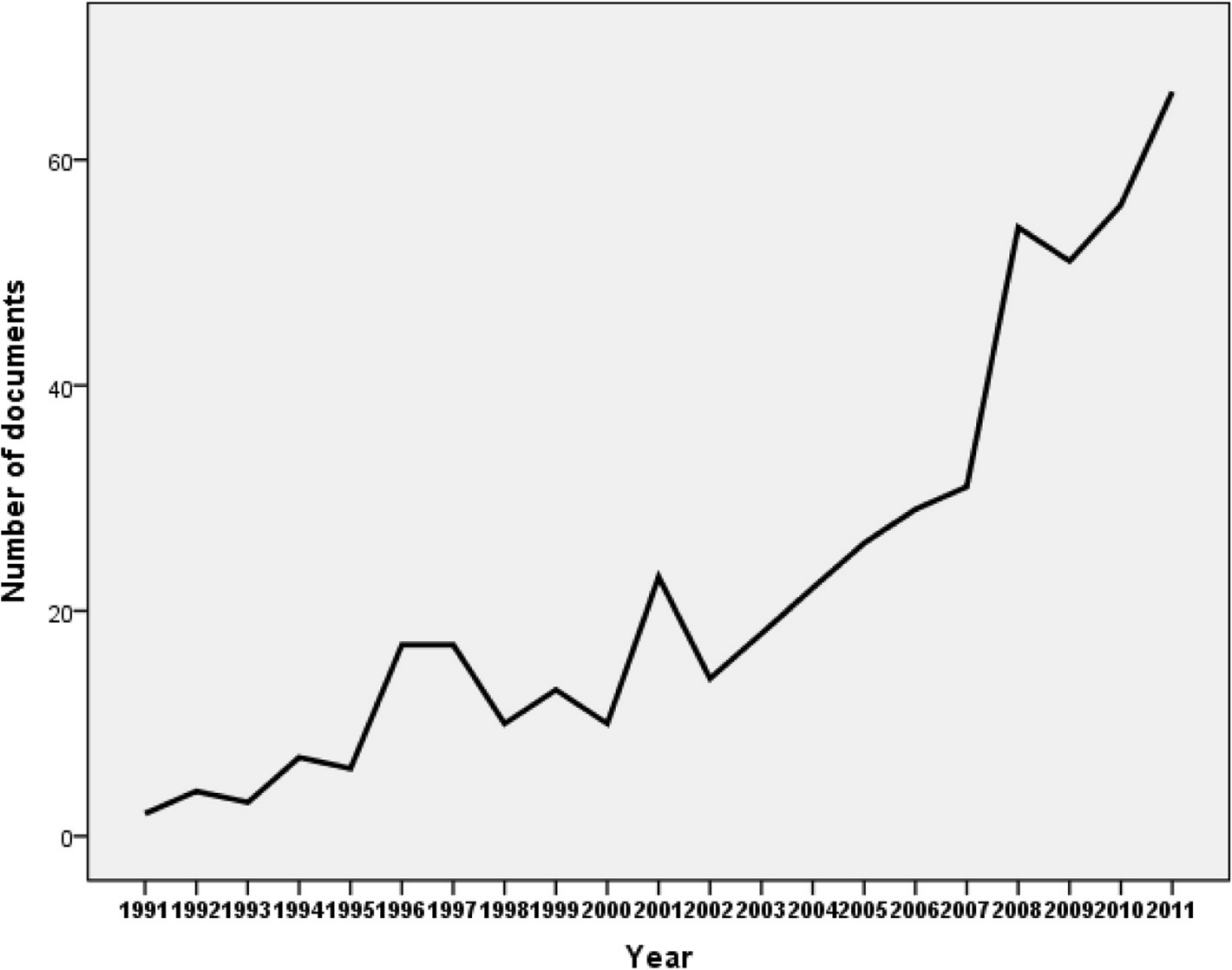
Growth of epidemiology-related obesity documents in Arab countries.

Research areas of obesity documents published from Arab countries were mainly in general/internal medicine, followed by endocrinology/metabolism and nutrition. Top 10 research areas are shown in Table 2. Analysis of research areas showed that endocrinology/metabolism and nutrition research areas had the highest increase with time, particularly in the past 5 years (Figure 2). On the other hand, research areas pertaining to cardiovascular or public/environmental/occupational health gained lesser momentum with time. Further analysis of the 1,121 obesity documents using “epidemiology” or “prevalence” key words yielded a total of 479 documents. Figure 3 shows the number of epidemiology-related documents as a function of time (Figure 3). Again, KSA 158 (32.99) had the highest number of epidemiology-related obesity documents and this constitutes approximately one third of epidemiology-related documents about obesity from Arab countries. However, Kuwait (19.38 documents per one million inhabitants) had the highest number of documents when data were adjusted per one million inhabitants, followed, again, by Bahrain (18.97) and Qatar (13.6) (Table 3).

**Table 2:** Research areas of published obesity documents from Arab countries

**SCR**^**a**^	**Research area**	**Number (%)**
		**n = 1,121**
**1**^**st**^	General Internal Medicine	232 (20.7)
**2**^**nd**^	Endocrinology Metabolism	191 (17.04)
**3**^**rd**^	Nutrition Dietetics	155 (13.83)
**4**^**th**^	Public Environmental Occupational Health	91 (8.12)
**5**^**th**^	Cardiovascular System Cardiology	62 (5.53)
**5**^**th**^	Surgery	62 (5.53)
**7**^**th**^	Research Experimental Medicine	56 (5.0)
**8**^**th**^	Pediatrics	49 (4.37)
**9**^**th**^	Pharmacology Pharmacy	38 (3.39)
**10**^**th**^	Biochemistry Molecular Biology	36 (3.21)

SCR, Standard Competition Ranking.

^a^Equal research areas have the same ranking number and then a gap is left in the ranking numbers.

**Table 3:** Number of epidemiology-related obesity documents stratified by country and standardized by million inhabitants

**Country**	**Number of epidemiology-related documents**	**Number of epidemiology-related documents per one million inhabitants**
	**n = 479 (%)**^**a**^	
KSA	158 (32.99)	5.58
Kuwait	63 (13.15)	19.38
UAE	50 (10.44)	5.43
Egypt	48 (10.02)	0.59
Tunisia	43 (8.98)	3.99
Jordan	31 (6.47)	4.90
Qatar	27 (5.64)	13.16
Lebanon	26 (5.43)	5.87
Bahrain	25 (5.22)	18.97
Morocco	22 (4.59)	0.62
Oman	20 (4.18)	6.03
Yemen	7 (1.46)	0.29
Algeria	6 (1.25)	0.16
Sudan	5 (1.04)	0.13
Iraq	4 (0.84)	0.12
Libya	4 (0.84)	0.65
Syria	2 (0.42)	0.09

^a^Total exceeds 100% because of overlap in some documents among more than one Arab country.

Collaboration between Arab countries and non-Arab countries in obesity research and publication was evident. Countries whose researchers collaborated most with investigators in the Arab world include the USA (121; 10.79%), followed by France (97; 8.65%) and England (80; 7.14%).

Table 4 lists the top 10 journals in which obesity-related documents were authored or co-authored by investigators from Arab countries. Approximately 10% of documents about obesity published from Arab countries were published in the *Saudi Medical Journal*, which is a medical journal with a wide scope. Of the top 10 journals in which documents about obesity were published from the Arab world, 2 journals (*Obesity Reviews* and *International Journal of Obesity*) had an IF > 5. Of the top 10 journals, 2 journals were based and published from KSA. Interest of Arab gulf researchers in obesity research is evident in the top 10 Arabic institutions involved in obesity research (Table 5). The most productive institution was King Saud University (140; 12.49%) in KSA. Except for the Saudi Ministry of Health, all institutions in the top 10 list were academic institutions; 4 of the top 10 institutions were based on KSA and 2 were based in Egypt.

**Table 4:** Top 10 journals in which obesity documents from the 21 Arab countries were published

**SCR**^**a**^	**Journal**	**Number (%)**	**IF**^b^
		**n = 1,121**	
**1**^**st**^	Saudi Medical Journal	107 (9.55)	0.619
**2**^**nd**^	Annals of Saudi Medicine	58 (5.17)	1.103
**3**^**rd**^	Obesity Surgery	28 (2.50)	3.102
**4**^**th**^	International Journal of Obesity	21 (1.87)	5.221
**5**^**th**^	Medical Principles and Practice	18 (1.61)	0.963
**6**^**th**^	Diabetes Research and Clinical Practice	17 (1.52)	2.741
**7**^**th**^	Diabetes Metabolism	14 (1.25)	2.388
**7**^**th**^	Public Health Nutrition	14 (1.25)	2.250
**9**^**th**^	Annals of Nutrition and Metabolism	13 (1.16)	1.661
**10**^**th**^	Obesity Reviews	12 (1.07)	6.870

SCR, Standard Competition Ranking; NA, Not available; IF, Impact factor.

^a^Equal journals have the same ranking number, and then a gap is left in the ranking numbers.

^b^The impact factor was reported according to Institute for Scientific InformationJournal Citation Reports, 2012.

**Table 5:** Top 10 active institutions in obesity research from Arab countries

**SCR**^**a**^	**Institution**	**Number (%)**	**Address**
**1**^**st**^	King Saud University	140 (12.49)	KSA
**2**^**nd**^	Kuwait University	97 (8.65)	Kuwait
**3**^**rd**^	United Arab Emirates University	53 (4.73)	UAE
**4**^**th**^	American University of Beirut	42 (3.75)	Lebanon
**4**^**th**^	Cairo University	42 (3.75)	Egypt
**6**^**th**^	Ain Shams University	41 (3.66)	Egypt
**7**^**th**^	Ministry of Health	38 (3.39)	KSA
**8**^**th**^	King Faisal Specialist Hospital Research Center	35 (3.12)	KSA
**9**^**th**^	Jordan University of Science Technology	33 (2.94)	Jordan
**10**^**th**^	King Abdulaziz University	32 (2.86)	KSA

SCR, Standard Competition Ranking; KSA, Kingdom of Saudi Arabia; UAE, United Arab Emirates.

^a^Equal institutions have the same ranking number, and then a gap is left in the ranking numbers.

The total number of citations for obesity documents from the Arab world, at the time of data analysis (March 8^th^, 2014), was 13,343 with an average citation of 11.9 per document. The total citation of obesity documents from the Arab world excluding self-citation was 11,886. Of the 1,121 documents considered for the *h*-index, 44 had been cited at least 44 times at the time of data analysis. Compared with other non-Arab countries in the Middle East, the research productivity from the Arab countries was lesser than that from Turkey, higher than that from Iran, and close to that from Israel. The *h*-index of documents about obesity published from Arab countries was lower than that of Turkey and Israel but slightly higher than that from Iran (Table 6).

**Table 6:** Research about obesity from Arab countries compared with that from Turkey, Iran, and Israel

**Data/Country**	**Arab countries**	**Turkey**	**Israel**	**Iran**
Number of population in millions	400	60	8	80
Number of published research articles and review articles	1,121	1,322	1,039	504
Total number of citations	13,343	17,479	28,738	7,251
Average citation per document	11.9	13.22	27.66	14.37
*h*-index	44	52	78	40

## Discussion

Obesity is a leading cause of mortality and morbidity and all types of research in this field is required to promote better health at the individual and community levels. There is no doubt that a large amount of research has been carried out and published from the Arab world regarding obesity especially in recent years. This is a reflection of the global increase in the attention of health workers to obesity as a common and preventable risk factor for a wide range of endocrine and cardiovascular diseases. Efforts to combat obesity in Arab countries require a collaboration in the field of obesity research, particularly with epidemiological and genetic research. Ongoing research requires periodical quantitative and qualitative assessment so that gaps in the field of obesity could be bridged among different countries and among different ethnic groups. In our study, we carried out a bibliometric analysis of obesity research output from Arab countries using WoS, which is a rich research database with powerful citation analysis services [13,22-26]. Analysis of research output from a particular country is an image of its research activity and capacity. Of course, the research activity and capacity of a particular country depends on several factors including the national income and the size of the population. In the case of Arab countries with approximately 400 million population and huge resources, excellence in research is a must. To the best of the authors’ knowledge, our study is the first article to analyze the quantity and quality of research about obesity specifically from the Arab world. Our study showed that some Arab countries, such as KSA and Egypt, clearly had a higher research output than the remaining Arab countries.

In our study, a total of 1,121 original research articles and reviews from Arab countries related to obesity were retrieved using WoS database. The actual number of documents might be higher than this given that some journals in which Arab researchers have published about obesity were not indexed in WoS. Despite that, our study reflects a close approximation of obesity research activity in the Arab countries published in international and reputable journals. Finally, the data obtained in our study will serve as a baseline data for evaluation of future research activities and for comparative purposes with other non-Arab countries.

Our results indicated that Kuwait ranked first in research productivity regarding obesity research. A study investigating the prevalence of overweight and obesity among adolescents in seven Arab countries found that Kuwaiti adolescents showed the highest prevalence of obesity for both males (34.8%) and females (20.6%) [6]. Another review article indicated that the high prevalence of obesity and overweight in Arabic region is possibly due to cultural and nutritional transitions [26,27]. Several studies reported that the prevalence rates of obesity are high. A recent review study about obesity in Kuwait reported that the prevalence of obesity ranged from 24% to 48% overall and, in adults over 50 years, it was greater than 52% [28]; further, they found that trends of obesity showed an increase in prevalence between 1980 and 2009. The findings that Bahrain ranked second in number of documents per million about obesity was also expected. A study from Bahrain found that the prevalence rates for obesity in Bahrain are higher than predicted and are increasing at a higher rate than the global average; this mirrors the alarming increase in the prevalence of Type 2 Diabetes Mellitus in Bahrain [27]. The increased prevalence of obesity in Arab gulf countries seems to be associated with increased prevalence of impaired glucose, hypertension, and dyslipidemia among people in Arab gulf countries [29]. Several published articles from Arab gulf countries have called for national policies to combat and face the escalating problem of obesity and its associated risks [30-33]. Thus, it is not surprising that Arab gulf countries had high obesity-related research productivity. Obesity and diabetes mellitus have reached alarming points in Arab Gulf countries and no wonder that researchers from these countries have high research activity regarding obesity. Furthermore, the relatively good economy of Arab gulf countries participated in pushing medical research activity forward. In some Arab countries, such as KSA, the Ministry of Health was actively involved in obesity research. This suggests that health policy makers are aware of the dangers of this national health condition and its economic and health impact. The low research activity about obesity in some Arab countries could be attributed to political and military conflict along with a lack of democracy and governmental funding.

Our results showed that interest in obesity research in Arab countries showed a dramatic increase over the last decade, probably due to serious epidemiological studies published in late 1990s regarding diabetes mellitus, hypertension, and chronic kidney diseases [34-37]. Arab countries have surpassed Israel and Iran in obesity-related research activity. This should not be surprising given that reported prevalence of adult obesity in Arab countries is higher than that in Israel or Iran. However, the quality of obesity research from Arab countries was not higher than that produced from neighboring non-Arab countries as measured by citation analysis. This means that, although attention to obesity research is growing, the type and quality of obesity publications is not as those from Israel or Turkey. Citation is a key indicator of research quality and researchers need to be aware of the mechanisms that might enhance the citation of published articles, such as self-citation, whenever possible [38]. Highly cited articles positively contribute to the *h*-index of the individual author and to the institution and country [25]. One possible reason for differences in average citation and *h*-index between publications of Arab countries and those from other non-Arab countries is the IF of journals in which researchers publish their articles. It is believed that articles in high IF journals have higher chance of being cited, although this is not guaranteed [24].

Our results showed that authors from Arab countries collaborated mainly with authors from USA in obesity research. International collaboration is believed to increase the quantity and quality of research activity [39]. Other studies indicated that international collaboration can increase the visibility of scientific publications from a particular country [40]. Furthermore, international collaboration in research helps capacity building in developing countries and makes national problems of developing countries more observable [41].

Our study has few limitations that need to be mentioned, most of which are the same as those of studies performed in other biomedical fields [18,42,43]. First of all, we used the WoS database; therefore, articles published in journals outside the WoS database were not included. Another limitation is that some international journals do not recognize countries like Palestine as a separate country and publications from Palestine may be affiliated with Israel. Therefore, some publications from Israel were actually from Palestine. Finally, it should be noted that research output for certain institutions could have been underestimated because due to differences in writing their English names in various articles; therefore, such institutions might have two or more profiles in WoS.

## Conclusions

The present data reveals a good late momentum for obesity research from the Arab world, particularly from Arab gulf countries. However, the quality of research about obesity from Arab countries was lower than that from non-Arab countries. Academic institutions in Arab countries are advised to strengthen research collaboration with international researchers and institutions in which obesity research has evolved.

## Abbreviations

IF: Impact factor; IRB: Institutional Review Board; KSA: Kingdom of Saudi Arabia; WoS: Web of Science.

## Competing interests

The authors declare that they have no competing interests.

## Authors’ contributions

All authors were involved in drafting the article and all authors approved the final version to be submitted for publication. All authors have added an intellectual significant value to the manuscript.
